# Membrane depolarization-triggered responsive diversification leads to antibiotic tolerance

**DOI:** 10.15698/mic2015.08.220

**Published:** 2015-07-24

**Authors:** Natalie Verstraeten, Wouter J. Knapen, Maarten Fauvart, Jan Michiels

**Affiliations:** 1 Centre of Microbial and Plant Genetics, KU Leuven - University of Leuven, 3001 Leuven, Belgium.

**Keywords:** Obg, ObgE, CgtA, YhbZ, persistence, antibiotic tolerance, (p)ppGpp, HokB, toxin antitoxin, responsive diversification, membrane depolarization

## Abstract

Bacterial populations are known to harbor a small fraction of so-called persister cells that have the remarkable ability to survive treatment with very high doses of antibiotics. Recent studies underscore the importance of persistence in chronic infections, yet the nature of persisters remains poorly understood. We recently showed that the universally conserved GTPase Obg modulates persistence via a (p)ppGpp-dependent mechanism that proceeds through expression of hokB. HokB is a membrane-bound toxin that causes the membrane potential to collapse. The resulting drop in cellular energy levels triggers a switch to the persistent state, yielding protection from antibiotic attack. Obg-mediated persistence is conserved in the human pathogen *Pseudomonas aeruginosa*, making Obg a promising target for therapies directed against bacterial persistence.

Over seven decades ago, Joseph Bigger observed that a small number of cells within a *Staphylococcus pyogenes* population was not killed by penicillin treatment. Curiously, these cells were not resistant to the antibiotic at hand, as they gave rise to a new population that again showed susceptibility towards penicillin. Bigger appropriately named the persevering cells persisters. Persister cells are now known to withstand antibiotic therapy and to resuscitate when the therapy is stopped, thereby contributing to the chronic nature of many infections. The development of novel techniques to study small fractions of cells that only transiently display multidrug tolerance, along with the recognition of their clinical importance, has substantially fueled persistence research. At present, it is clear that cells can switch to the persistent state as a result of stochastic expression of persister genes. In addition, persistence can develop as a result of environmental stimuli.

The essential and conserved P-loop GTPase Obg has previously been implicated in the coordination of many cellular functions including ribosome biogenesis, DNA replication, and several stress responses. In addition, it has been dubbed a cell cycle sentinel, tuning its activities according to the cell’s energy status in order to enhance bacterial survival in hostile environments. These previously reported properties made Obg an attractive candidate to regulate entry into the persistent state. We assessed this by both overexpressing and depleting Obg in the model bacterium *Escherichia coli*. Our results show a direct and strong correlation between cellular Obg concentrations and persister levels of *E. coli *populations. Using fluorescence-activated cell sorting (FACS), we additionally demonstrated at the single-cell level that high Obg levels, originating from stochastic fluctuations in *obg *gene expression, significantly increase the likelihood of surviving lethal antibiotic treatment. Combined, our results indicate that naturally occurring cell-to-cell variations in expression of *obg *contribute to persistence.

As well as noisy gene expression, environmental triggers have previously been shown to promote persistence development. Earlier research revealed an important role for the stringent response to nutrient limitation in the emergence of persistence. In *E. coli*, nutrient limitation is signaled by the alarmone (p)ppGpp that is produced by RelA and SpoT and broken down by SpoT. Interestingly, Obg is known to associate with both (p)ppGpp and SpoT. Using both mutants defective in the stringent response and mutants producing intermediate levels of (p)ppGpp, we showed that Obg-mediated persistence is dependent on (p)ppGpp but not on SpoT. In addition to stochastic fluctuations in Obg levels, the newly-unraveled persistence mechanism therefore also depends on environmental stimuli and thus represents an example of responsive diversification. At present, it is not known whether (p)ppGpp binding to Obg is required to induce persistence. This association might cause conformational changes that redirect Obg’s activities. In an alternative scenario, (p)ppGpp might affect a hitherto unidentified factor that, along with Obg, drives persistence.

To identify potential effectors of Obg-mediated persistence, we performed a transcriptome analysis, comparing Obg overexpression and depletion strains. Strikingly, among the differentially expressed genes was *hokB*. *hokB *is part of a chromosomal toxin-antitoxin module and encodes a small peptide that localizes to the bacterial membrane. Hok toxins were first identified on plasmids where they constitute a maintenance mechanism. Hok translation is tightly regulated by its cognate antitoxin *sok* and an additional regulator *mok*. However, little is known about transcriptional control of chromosomal *hok-sok *modules. Our results indicate that Obg is a regulator of *hokB *gene expression, as we observe a direct correlation between cellular Obg concentrations and *hokB *mRNA levels, both at population level and at single-cell level. Possibly, Obg could interfere with post-transcriptional stabilization of *hokB *mRNA. Alternatively, Obg could directly or indirectly activate *hokB *transcription. However, Obg is not known as a transcription factor and the protein has never been reported to affect gene expression in a direct manner. Therefore, indirect regulation of *hokB *transcription via an as yet to be identified effector is a more plausible scenario. Proteins whose expression is regulated by (p)ppGpp are among the likely effectors, as Obg does not induce *hokB *expression in a strain lacking (p)ppGpp.

In addition to providing a first clue as to the regulation of *hokB *transcription, our data show that Obg-mediated persistence is dependent on HokB. Overexpression of *obg *no longer increases persistence in a *hokB *knockout strain. In addition, ectopic overexpression of *hokB *increases persistence significantly. These findings are indicative of a model in which Obg, through transcriptional activation of *hokB, *affects persistence. Previously, it was suggested that Hok toxins cause the transmembrane potential to collapse. We confirmed this using a fluorescent dye that only enters depolarized cells. Overexpression of Obg also results in membrane depolarization. On the other hand, artificially increasing the membrane potential using the photoprotein proteorhodopsin or the metabolite mannitol completely abolishes Obg- or HokB-mediated persistence. Combined, these results strongly suggest that the newly-discovered persistence mechanism proceeds through membrane depolarization, leading to a decrease in cellular energy levels and the induction of the persistent state. Of note, depletion of Obg in a strain deleted for *hokB *reduces persistence, suggesting the existence of at least one additional pathway through which Obg affects persistence. In addition, it remains to be investigated how HokB affects the membrane potential. Reminiscent of previously described toxins TisB and GhoT, HokB might form pores that cause membrane permeability. Alternatively, as has been suggested previously, HokB might interact with specific membrane-bound entities. Candidates of interest in this respect are components of the respiratory chain, inhibition of which could interfere with electron transport and proton pumping, eventually leading to a collapse of the membrane potential.

In summary, we have revealed a signaling cascade through which Obg activates *hokB *expression in a (p)ppGpp-dependent manner, ultimately leading to membrane depolarization and persistence (Figure 1). Of note, we have shown that Obg-mediated persistence is also found in the human pathogen *P. aeruginosa, *suggesting that it represents a conserved mechanism of antibiotic tolerance. The decreased persister fraction in an Obg depletion strain that we report here makes Obg a promising target for therapies directed against bacterial persistence. Further elucidation of this pathway will lead to a better understanding of the persistence phenomenon, which may lead to the development of new drugs to combat chronic infections.

**Figure 1:**
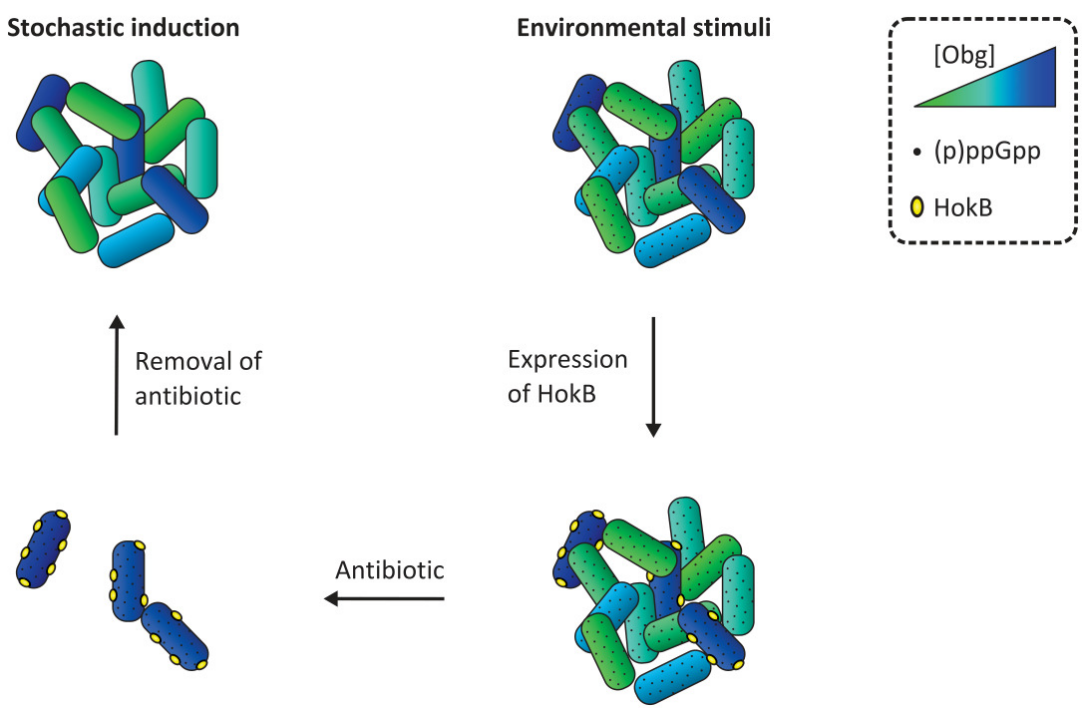
FIGURE 1: A model for Obg-mediated persistence. Stochastic fluctuations in Obg concentrations drive expression of HokB in a (p)ppGpp-dependent manner. HokB protects the cells from antibiotic attack by reducing the membrane potential. Persister cells surviving antibiotic treatment give rise to a new population. See text for details.

